# Sleep Disorders and Quality of Life in Patients With Cancer: Prospective Observational Study of the Rafael Institute

**DOI:** 10.2196/37371

**Published:** 2022-11-24

**Authors:** Nathaniel Scher, Liath Guetta, Clément Draghi, Safia Yahiaoui, Mathilde Terzioglu, Emilie Butaye, Kathy Henriques, Marie Alavoine, Ayala Elharar, Andre Guetta, Alain Toledano

**Affiliations:** 1 Integrative Medicine Rafael Institute Levallois-Perret France; 2 Hartmann Oncology Radiotherapy Group Hartmann Radiotherapy Institute Levallois Perret France; 3 Department of Radiotherapy Institut Salah Azaiz Tunis Tunisia; 4 Department of Integrative Medicine National Conservatory of Arts and Crafts Paris France

**Keywords:** cancer, sleep disorder, sleep, fatigue, nocturnal, oncology, cancer care, patient-centred approach, patient-centered, personalized, personalization, customized, customization, care plan, quality of life, mood, pain, cancer treatment, overweight, obese, hormone therapy, breast, prostate

## Abstract

**Background:**

Sleep disorders are a common occurrence in the general population. Yet today, it is clearly agreed that sleep disorders represent both a cancer risk factor and a biological consequence of the of the activation of the immuno-inflammatory system induced by cancer itself.

**Objective:**

The aim of this study was to assess the impact of sleep disorders on quality of life and identify the type of disorder and its causes in order to offer an adapted and personalized care plan.

**Methods:**

In a survey completed during the COVID-19 lockdown, 2000 hours of interviews were collected by remote consultations. During these calls, we administered a sleep questionnaire. This questionnaire was inspired by the STOP-BANG questionnaire and enquired about 6 items. The demographic details of each patient (eg, age and sex), the nature of the pathology, their past treatments, the ongoing cancer treatment, the mood, whether or not the patient is anxious or depressed, and the use of sleeping drug pills were analyzed. A univariate analysis was performed according to the presence or absence of fatigue. Chi-square test was applied to assess possible differences of variables’ link to sleep disturbance between patients complaining of fatigue and those without fatigue. The same test was then used to analyze patients on hormone therapy and those with no hormone therapy for 2 types of cancer—breast cancer and prostate cancer.

**Results:**

A total of 905 patients were prospectively included in this study. The average age was 66.7 (5 SD) years, and 606 (67%) patients were women; 142 patients declared being overweight. Breast cancer was the most frequently reported cancer. Nocturnal awakening was reported by 70% (n=633), fatigue by 50% (n=452), difficulty falling asleep by 38% (n=343), snoring reported by an independent observer in 38% (n=343), and apnea reported by an independent observer in 9% (n=81) of the patients. The univariate analysis showed that the feeling of tiredness was significantly greater in patients reporting difficulty falling asleep (*P*≥.99), pain (*P*<.001), and frequent awakening (*P*<.001), as well as in patients who were not receiving cancer treatment (*P*<.001). The univariate analysis showed that patients who were receiving breast cancer treatment and were under hormone therapy reported difficulty falling asleep (*P*=.04) and pain (*P*=.05). In a univariate analysis of patients treated for prostate cancer, being overweight was the only factor reported that had a statistically significant value.

**Conclusions:**

Our preliminary data support and are consistent with data in the literature regarding the importance of sleep disorders in oncology. This justifies the usefulness of a diagnosis and early treatment of sleep disorders in patients with cancer. The Rafael Institute sleep observatory will enable patients to be identified and treated.

## Introduction

Sleep disorders are a common occurrence in the general population. The most common sleep disorders are the following: insomnia (with a prevalence of 6% to 20%) [1]; rapid eye movement sleep behavior disorder (with a prevalence of 3% to 10%); restless legs syndrome (5% to 11%); periodic limb movements during sleep (up to 30%); obstructive sleep apnea (9% to 38%); and circadian rhythm sleep-wake disorders (3% to 10%) [2-4]. The prevalence of various types of sleep disorders in patients with cancer has not been widely investigated, as sleep disorders are often not assessed in accordance with the International Classification of Sleep Disorders Third Edition in this group of patients [5]; yet today, it is clearly agreed that sleep disorders represent both a cancer risk factor and a biological consequence of the activation of the immuno-inflammatory system induced by cancer [6]. The importance of recognizing and treating sleep disorders in an appropriate manner is crucial if we consider that they can persist for several years after the cessation of antitumor treatment (eg, chemotherapy, radiotherapy, and hormonal therapy) and be a potential cause of comorbidity and impaired quality of life [7,8]. During the last decade, several authors have been particularly interested in sleep disorders and their harmful effects on each stage of cancer treatment for the patient [9,10]. It is therefore essential to acquire a better understanding of these disorders for which treatments offer promising therapeutic prospects in oncology, the aim being to enhance patients’ adhesion to stressful treatments and improve their quality of life.

The Rafael Institute, a postcancer institute, is a center for integrative medicine. Nearly 80 medical and paramedical care staff accompany patients during and after cancer treatments to improve their resilience. During the past 16 months, 1350 patients have received over 11,400 care treatments. In the context of personalized care plans oriented toward nutrition, emotional well-being, physical activity, and general well-being, a sleep center was inaugurated in the Rafael Institute in March 2020. The aim of our study was to assess the impact of sleep disorders on quality of life and identify the type of disorder and its causes in order to offer an adapted and personalized care plan.

## Methods

### Procedure

In a survey completed during the COVID-19 lockdown, 2000 hours of interviews were collected by remote consultations. During the lockdown, Rafael Institute caregivers made calls to maintain a link with confined patients. During these calls, we administered a sleep questionnaire. All of our patients were first contacted by email. The “RAFAËL SLEEP” questionnaire was created for this purpose. This questionnaire, inspired by the STOP-BANG questionnaire enquired about the following 6 items concerning sleep disorders: BMI, diurnal fatigue, difficulty falling asleep, nocturnal awakening, as well as snoring and apnea observed by an independent observer. Patients answering at least 2 questions in the affirmative were included in the study. The demographic details of each patient (eg, age and sex), the nature of the pathology, the treatment already received, the ongoing cancer treatment, the mood, whether or not the patient is anxious or depressed, and the use of sleeping drug pills were analyzed. A univariate analysis was performed according to the presence or absence of fatigue.

Chi-square test was applied to assess possible differences of variables link to sleep disturbance between patients complaining of fatigue and those without fatigue. The same test was then used to analyze patients on hormone therapy and those with no hormone therapy for 2 types of cancer—breast cancer and prostate cancer. R (version 4.1.2; R Foundation for Statistical Computing) was used to conduct all the statistical analyses in this study, and a *P*<.05 (two-tailed *P* value test) was considered as statistically significant.

### Ethics Approval

This observational prospective study was approved by the institutional review board of Rafael Institute and the ethical committee of Hartmann Oncology Radiotherapy Group. Informed consent was obtained from each participant. All necessary measures to safeguard participants’ anonymity and confidentiality of information were respected.

## Results

**Figure 1:**
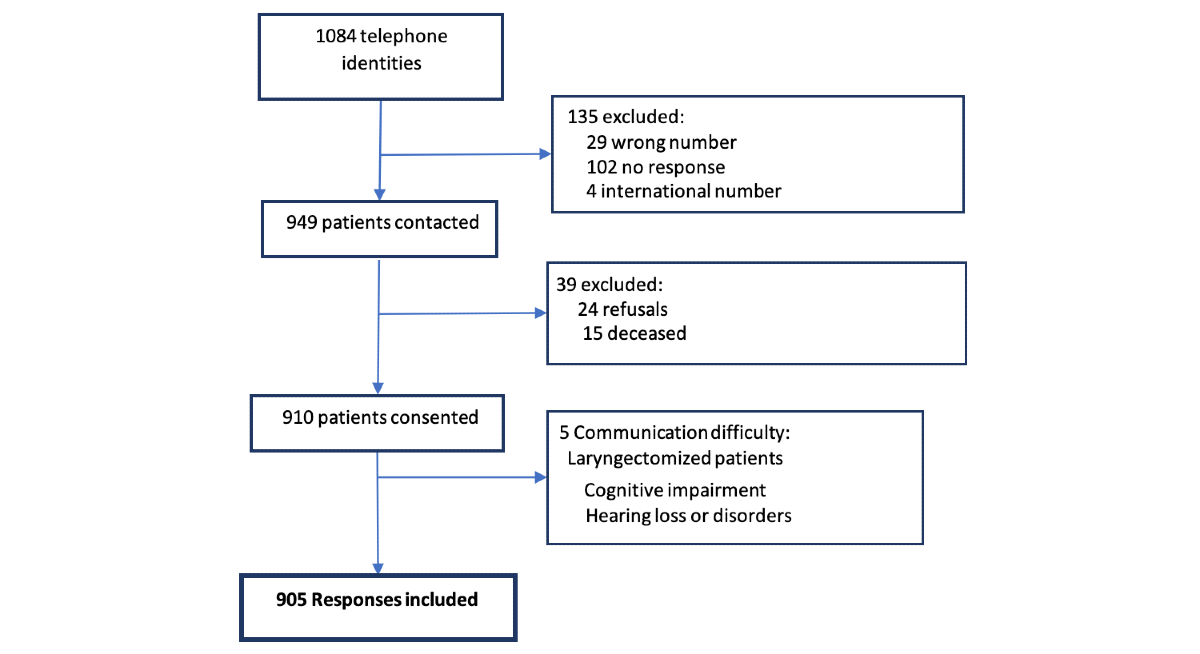
Flowchart of patients included in the study.

A total of 1084 phone numbers were initially selected for the telephone interviews on sleep disorders; 135 were excluded (29 refusals, 102 telephone errors, 15 deceased patients, and 5 for communication difficulty). We were able to include the responses of 905 patients in our study. The participation rate was 95% ( Figure 1). The average age was 66.7 (SD 5) years, and 606 (67%) were women; 142 patients declared being overweight. Breast cancer was the most frequently reported type of cancer in our population, with a prevalence of 60% (Table 1).

The sleep-affecting criteria that were assessed were fatigue, difficulty falling asleep, nocturnal awakening, as well as snoring and apnea reported by an independent observer. With respect to these criteria, nocturnal awakening was reported by 70% (n=635), fatigue by 50% (n=452), difficulty falling asleep by 38% (n=343), snoring reported by an independent observer in 38% (n=343), and apnea reported by an independent observer in 9% (n=81) of the patients. The results of the assessment showed that 18% (n=163) of patients responding to the questionnaire satisfied 2 sleep disorder criteria; 24% (n=217) fulfilled 3 sleep criteria; 13% (n=118) fulfilled 4 sleep criteria; and 5% (n=45) related to 5 criteria. A total of 50% (n=452) of patients in the observation group declared that they used sleeping pills. No significant difference was noted in the declaration of sleep disorders, whether or not the patients declared taking insomnia treatment.

The univariate analysis showed that the feeling of tiredness was significantly greater in patients reporting difficulty falling asleep (*P*≥.99), pain (*P*<.001), and frequent awakening (*P*<.001), as well as in patients who were not receiving cancer treatment (*P*<.001; Table 2). The univariate analysis showed that patients who were receiving breast cancer treatment and were under hormone therapy essentially reported difficulty falling asleep (*P*=.04) and pain (*P*=.05; Table 3). In a univariate analysis of patients treated for prostate cancer, being overweight was the only factor reported that had a statistically significant value (Table 4).

**Table 1:** Patient characteristics (N=905).

Characteristics		Values, n (%)
**Sex**		
	Women	687 (76)
	Men	218 (24)
**Localization**		
	Breast	539 (60)
	Prostate	107 (12)
	Other	67 (7.4)
	Head and neck	63 (7)
	Lung	43 (4.8)
	Colorectal	39 (4.3)
	Uterus	17 (1.9)
	Pancreas	16 (1.8)
	Ovary	10 (1.1)
**Day fatigue**		
	No	454 (50)
	Yes	447 (50)
**Apnea**		
	No	813 (90)
	Yes	88 (9.8)
**Appetite**		
	Fairly	463 (51)
	A lot	261 (29)
	A little	129 (14)
	Not at all	48 (5.3)
**Difficulties falling asleep**		
	No	552 (61)
	Yes	349 (39)
**Pain**		
	No pain at all	349 (39)
	A little	291 (32)
	Fairly	165 (18)
	A lot	96 (11)
**Hormonal therapy**		
	No	604 (67)
	Yes	297 (33)
**Frequent awakening**		
	No	266 (30)
	Yes	635 (70)
**Snoring**		
	No	548 (61)
	Yes	353 (39)
**Overweight**		
	No	659 (73)
	Yes	242 (27)
**Antitumor treatment completed**		
	No	418 (46)
	Yes	483 (54)

**Table 2:** Univariate analysis of fatigue. Italicized *P* values are statistically significant.

Characteristics			Absence of diurnal fatigue (n=454)		Presence of diurnal fatigue (n=447)		Total participants, n		*P* value	
Anxiety, mean (SD)			4.94 (2.56)		5.57 (2.50)		901		*<.001*	
Physical form, mean (SD)			7.44 (1.55)		6.21 (1.77)		901		*<.001*	
Moral, mean (SD)			7.62 (1.70)		6.63 (1.99)		901		*<.001*	
Quality of life, mean (SD)			7.72 (1.46)		6.90 (1.86)		901		*<.001*	
**Apnea, n (%)**										*≥.99*
	Absent	424 (93)		389 (87)		813				
	Present	30 (6.6)		58 (13)		88				
**Appetite, n (%)**										*<.001*
	Fairly	245 (54)		218 (49)		463				
	A lot	159 (35)		102 (23)		261				
	A little	43 (9.5)		86 (19)		129				
	Not at all	7 (1.5)		41 (9.2)		48				
**Difficulty** **getting** **to** **sleep, n (%)**										*≥.99*
	No	302 (67)		250 (56)		552				
	Yes	152 (33)		197 (44)		349				
**Pain, n (%)**										*<.001*
	Not at all	226 (50)		123 (28)		349				
	A little	139 (31)		152 (34)		291				
	Fairly	63 (14)		102 (23)		165				
	A lot	26 (5.7)		70 (16)		96				
**Frequent** **awakening, n (%)**										*<.001*
	No	178 (39)		88 (20)		266				
	Yes	276 (61)		359 (80)		635				
**Snoring, n (%)**										.6
	No	280 (62)		268 (60)		548				
	Yes	174 (38)		179 (40)		353				
**Sex, n (%)**										.45
	Woman	351 (77)		336 (75)		687				
	Man	103 (23)		111 (25)		214				
**Overweight, n (%)**										.07
	No	344 (76)		315 (70)		659				
	Yes	110 (24)		132 (30)		242				
**Treatment** **finished**										*≥.99*
	No	187 (41)		231 (52)		418				
	Yes	267 (59)		216 (48)		483				

**Table 3:** Univariate analysis of hormone therapy in patients treated for breast cancer.

Characteristics			No hormone therapy (n=278)		Hormone therapy (n=261)		Total number, n		*P* value
Anxiety, mean (SD)			5.28 (2.58)		5.70 (2.50)		539		.06
**Sleep disorders, n (%)**									.04
	Absent	178 (64)		145 (56)		323			
	Present	100 (36)		116 (44)		216			
**Pain, n (%)**									.05
	A little	109 (39)		82 (31)		191			
	Not at all	97 (35)		83 (32)		180			
	Fairly	46 (17)		61 (23)		107			
	A lot	26 (9.4)		35 (13)		61			
**Frequent** **awakening, n (%)**									.22
	Absent	99 (36)		80 (31)		179			
	Present	179 (64)		181 (69)		360			
**Snoring, n (%)**									
	Absent	180 (65)		164 (63)		344		.64	
	Present	98 (35)		97 (37)		195			
**Overweight, n (%)**									.13
	Absent	188 (68)		192 (74)		380			
	Present	90 (32)		69 (26)		159			

**Table 4:** Univariate analysis of hormone therapy in patients treated for prostate cancer. Italicized *P* values are statistically significant.

Characteristics		No hormone therapy (n=78), n (%)		Hormone therapy (n=29), n (%)		Total participants, n		*P* value	
**Apnea**									.19
	No	63 (81)	20 (69)		83				
	Yes	15 (19)	9 (31)		24				
**Difficulty** **getting** **to** **sleep**									.71
	No	54 (69)	19 (66)		73				
	Yes	24 (31%)	10 (34)		34				
**Pain**									>.99
	Not at all	45 (58%)	17 (59)		62				
	A little	20 (26)	6 (21)		26				
	Fairly	7 (9)	5 (17)		12				
	A lot	6 (7.7)	1 (3.4)		7				
**Frequent** **awakening**									.43
	No	19 (24)	5 (17)		24				
	Yes	59 (7)	24 (83)		83				
**Snoring**									.45
	No	36 (46)	11 (38)		47				
	Yes	42 (54)	18 (62)		60				
**Overweight**									*.04*
	No	59 (76)	16 (55)		75				
	Yes	19 (24)	13 (45)		32				
**Treatment** **finished**									*<.001*
	No	20 (26)	21 (72)		41				
	Yes	58 (74)	8 (28)		66				

## Discussion

Nearly one third of French adults have sleeping problems, such as sleep deficit, trouble getting to sleep or staying asleep, disturbance of the circadian rhythm, or respiratory problems such as obstructive apnea syndrome. The effects of sleep on immune, hormonal, cardiovascular, and neurocognitive functions are well established. An association between sleep disorders and the onset or progression of many cancers has now been suggested [11]. Recent epidemiological studies report a close link between cancer and sleep apnea syndrome [12-14] or circadian rhythm sleep disorders [15-18]. Working night shifts significantly increases the risk of cancer, particularly of prostate, colon, and breast cancer [11]. The disruption of sleep-wake circadian rhythms is an independent prognostic survival factor for patients with specific types of metastatic cancers [19-21]. The dysregulation of several dozen molecular signaling pathways that control the circadian rhythm may be involved in the carcinogenesis process [22,23].

The identification and correction of circadian rhythm sleep disturbances may prevent cancers or slow their progression [24]. Sleep apnea syndrome has been particularly well studied and may be strongly involved in mortality not only of cardiovascular origin but also mortality from all other causes, including cancer [25]. This high cancer mortality rate may be more specifically related to the apnea-hypopnea index [26,27]. An index of 30, for instance, increases the relative cancer mortality risk by a factor of 4.8. This is predominantly underpinned by intermittent hypoxia-induced tumor growth as it has been demonstrated in animal models [28,29]. Sleep disorders occur in 30%-75% of newly diagnosed patients or patients who have recently undergone cancer treatment. This rate is twice of that in the general population. Patients complain of difficulties getting to sleep and staying asleep, with frequent and prolonged nocturnal awakening [30,31]. Patients report difficulties both before and after cancer treatments. Fatigue is also a major factor in this particular population, and recent data suggest that daytime fatigue is related to the sleep-wake cycle and to the quality and quantity of sleep obtained throughout the night. The high prevalence of insomnia and sleep disorders experienced by patients with cancer has been attributed to cancer treatments, in particular chemotherapy, their side effects, psychosocial factors (eg, anxiety, stress, and depression), and circadian rhythm disruptions. The initial descriptive results from our cohort of 905 patients with cancer provide preliminary data for future studies that will more specifically focus on characterizing patient profiles and proposing an overall specific care plan for sleep disorders in this patient population.

In this preliminary study, 60% (n=543) of patients satisfy at least two of the criteria surveyed in the questionnaire; this is consistent with the percentage reported in numerous other studies on sleep disorders and cancer. As suggested in several studies, the predominant symptom in patients with cancer appears to be nocturnal awakening, which is also consistent with our observation, with some 70% (n=633) of interviewed patients complaining of this symptom. Nocturnal awakening results in sleep fragmentation, which may be the main risk factor in increasing the prevalence of cancers and potentially the resistance to chemotherapy. This nocturnal awakening may be due to disruptions of sleep-wake circadian rhythms, but also due to obstructive apnea syndrome. In our study, family members of 38% (n=344) of patients reported snoring and 9% (n=81) reported apnea; 38% (n=344) of patients complained of difficulty falling asleep; this is insomnia during the first part of the night, and this percentage is the same as reported in other studies on cancer and insomnia. The COVID-19 pandemic had a significant impact on all aspects of daily life. Sleep patterns, sleep quality, as well as the diagnosis and management of sleep disorders were all profoundly affected. In this study, the patient interview was conducted during the COVID-19 lockdown, and the survey analyzed the patient’s mood. Fear and anxiety of potential infection in a patient with cancer, mandatory confinements, and quarantine procedures likely combined to increase sleep dysfunction, as shown in other published studies [32]. This study does not specify the cause of the sleep disorders and does not analyze the specific impact of COVID-19 on the patients included. Nevertheless, our sleep laboratory has moved toward a new practice model, minimizing physical contact with patients and developing remote consultations to maintain contact with patients. This new effective assessment method has made it possible to structure the assessment of sleep disorders.

Conclusions from this study should be tempered in light of some limitations. First, this is an observational cohort and data were collected by remote consultation, which may have missed some important information. Second, although every attempt was made to identify all sleep disorders, the sleep-affecting criteria were reported by an independent observer and from a sleep questionnaire; therefore, it is possible that some data about sleep disorders were missed, and this might as well have affected our conclusions. The average age of our cohort was 66.7 (SD 5) years, which also corresponds to the average age of patients with cancer in France. There are markedly more women than men (n=678, 76% vs n=218, 24%). This is explained by the fact that more women adhere to the paramedical care offered at the Rafael Institute. We will therefore extend the sleep observatory to all patients with cancer who have sleep disorders, irrespective of whether or not they are being treated at the Rafael Institute.

These results have supported us in our strategy of global patient care. This will involve all new patients who come for consultations to the Rafael Institute and complete the questionnaire. If the questionnaire is positive, the patient will be offered simple respiratory polygraphy to eliminate any potential sleep apnea syndrome. Polygraphy has been chosen over ambulatory polysomnography, which is the reference examination but is more difficult to set up in the context of daily life and costs more. In the case of positive polygraphy—namely more than 30 apnea episodes per hour—patients are given a standard treatment, that is, automatically controlled continuous positive airway pressure. Otherwise, a specialized sleep consultation is proposed, with the programming of continuous positive airway pressure, if necessary. The aim of this consultation is to assess the specific sleep disorder and propose a suitable treatment. This treatment consists essentially of cognitive and behavioral therapies, the use of a therapy such as sophrology, verbalization psychotherapy, meditation, or neurofeedback. The aim is to treat sleep disorders without recourse to benzodiazepines. In the study, we found that 50% (n=452) of patients used sleeping pills. This very high percentage together with awareness of the deleterious effects of benzodiazepines encourages us to favor nonmedical treatments. Our preliminary data support and are consistent with data in the literature regarding the importance of sleep disorders in oncology. This justifies the usefulness of a diagnosis and early treatment of sleep disorders in patients with cancer. The Rafael Institute sleep observatory will enable patients to be identified and treated. The RAFAËL SLEEP questionnaire used in this study enables patients with sleep disorders to be identified. It will be complemented by sleep, anxiety, and quality of life questionnaires validated by learned societies to propose correlation studies. This will allow us to assess care objectively.
